# The Effect of Preoperative Epicardial Adipose Tissue Thickness on Postoperative Morbidity and Mortality in Patients Undergoing Isolated Coronary Artery Bypass Grafting

**DOI:** 10.3390/jcm15062207

**Published:** 2026-03-13

**Authors:** Fulya Topuz, Ali Ahmet Arıkan, Sadan Yavuz, Oguz Omay, Ozgur Baris

**Affiliations:** 1 Department of Cardiovascular Surgery, Yalova Training and Research Hospital, Yalova 77200, Türkiye; 2 Clinic for Thoracic and Cardiovascular Surgery, Heart and Diabetes Center NRW (North Rhine Westphalia), University Hospital of the Ruhr-University, 32545 Bad Oeynhausen, Germany; dr_aarikan@hotmail.com; 3 Department of Cardiovascular Surgery, Kocaeli University Faculty of Medicine, Kocaeli 41380, Türkiye; sadanyavuz67@yahoo.com.tr (S.Y.); oguzomay@gmail.com (O.O.); drozgurbaris@gmail.com (O.B.)

**Keywords:** epicardial adipose tissue, coronary artery bypass grafting, postoperative complications, atrial fibrillation, acute kidney injury

## Abstract

**Background and Objective:** Despite advances in operative techniques and perioperative care, complications following coronary artery bypass grafting (CABG) remain an important cause of postoperative morbidity and organ dysfunction. This study aimed to evaluate the association between preoperative epicardial adipose tissue (EAT) thickness measured using computed tomography and postoperative morbidity and mortality in patients undergoing isolated CABG, and to explore whether EAT thickness may serve as a potential imaging-based risk marker for postoperative complications. **Materials and Methods:** The study was a retrospective single-center observational cohort study. Patients who underwent isolated coronary artery bypass grafting between 1 January 2019 and 2 January 2023, and had available preoperative computed tomography (CT) imaging were retrospectively reviewed. Epicardial adipose tissue thickness was measured on CT images at three predefined anatomical regions, yielding two parameters: total EAT thickness and right ventricular EAT thickness. Postoperative complications were evaluated using established definitions, with atrial fibrillation (AF) assessed according to European Society of Cardiology (ESC) criteria and acute kidney injury defined based on Kidney Disease: Improving Global Outcomes (KDIGO) guidelines. **Results:** Patients who developed postoperative complications after coronary artery bypass grafting tended to have thicker epicardial adipose tissue. Increased total epicardial adipose tissue thickness was associated with postoperative paroxysmal atrial fibrillation, whereas greater right ventricular epicardial adipose tissue thickness was associated with postoperative acute kidney injury. Multivariable analysis confirmed that both total and right ventricular epicardial adipose tissue thickness were independently associated with postoperative complications (total EAT: OR 1.74, 95% CI 1.10–2.76; right ventricular EAT: OR 2.03, 95% CI 1.31–3.15). ROC analysis showed modest discrimination for postoperative atrial fibrillation (AUC 0.69) and acceptable discrimination for acute kidney injury (AUC 0.79). No association was observed between epicardial adipose tissue measurements and postoperative mortality. **Conclusions:** Increased preoperative epicardial adipose tissue thickness was associated with several early postoperative complications following coronary artery bypass grafting, including atrial fibrillation, acute kidney injury, and in-hospital infection. Preoperative epicardial adipose tissue thickness measured by computed tomography may represent a potentially useful imaging-based risk marker for early postoperative complications following isolated CABG, although confirmation in larger prospective studies is required.

## 1. Introduction

Coronary artery disease remains one of the leading causes of morbidity and mortality worldwide. Despite well-established traditional cardiovascular risk factors, the occurrence of atherosclerosis in individuals without these factors has stimulated interest in identifying novel biomarkers. In recent years, epicardial adipose tissue has attracted increasing attention as a potential imaging-based marker for the early detection of atherosclerosis and assessment of disease severity [1].

Coronary artery bypass grafting (CABG) remains one of the most frequently employed treatment modalities for high-risk patients with complex cardiovascular disease [2]. Over the past several decades, the risk profile of patients undergoing isolated CABG has changed substantially, due to older age and a greater burden of preoperative comorbidities [3]. Despite improvements in survival and operative safety, postoperative complications continue to cause significant organ injury and morbidity in surgical patients [4]. In this context, surgical myocardial revascularization remains an effective and durable treatment for coronary artery disease; however, myocardial injury related to cardiopulmonary bypass and intraoperative ischaemia–reperfusion remains an important cause of postoperative morbidity [5,6].

Cardiac surgery performed with cardiopulmonary bypass (CPB) is associated with a pronounced systemic inflammatory response that contributes to the development of postoperative complications such as atrial fibrillation, infection, and organ dysfunction [7,8]. Postoperative AF is one of the most common complications following cardiac surgery, with an incidence of approximately 30% after CABG [9]. Recent studies have supported an association between postoperative AF and proinflammatory biomarkers [10]. Postoperative AF complicates postoperative management, prolongs hospital length of stay, and increases the risk of cerebral infarction and both short- and long-term mortality [11]. Therefore, the prophylactic use of perioperative amiodarone or beta- blockers has been recommended to reduce the risk of postoperative AF following cardiac surgery [12].

Renal hypoperfusion and inflammatory injury associated with CPB are important contributors to postoperative renal dysfunction [13]. Off-pump CABG techniques may reduce this risk by eliminating the use of cardiopulmonary bypass; however, an inflammatory response related to surgical trauma persists even in off-pump cardiac surgery [14].

Recognition of the importance of both the physiological and pathological roles of EAT on the coronary arteries has led to numerous studies aimed at elucidating the relationship between the development of coronary atherosclerosis and predisposing factors. Epicardial adipose tissue is known to be associated with the development of coronary artery disease. However, although several studies have evaluated epicardial adipose tissue in relation to cardiovascular outcomes, including postoperative atrial fibrillation, data specifically examining preoperative EAT thickness and its association with multiple postoperative complications following isolated CABG remain limited.

Predicting the risk of morbidity and mortality following cardiac surgery is critical for clinical management, and there is a continuing need for reliable predictors of early postoperative complications. In this context, EAT has attracted considerable attention as a metabolically active visceral fat depot capable of producing multiple bioactive molecules [1,15]. Under normal physiological conditions, epicardial adipose tissue exerts cardioprotective effects by secreting anti-atherogenic adipokines; however, an increased amount of EAT contributes to the development of atherosclerosis through the production of pro- inflammatory and proatherogenic molecules.

Epicardial adipose tissue is predominantly distributed along the atrioventricular and interventricular grooves, closely surrounding the coronary arteries and myocardium without a distinct fascial layer separating the coronary vessels from the myocardial surface [16]. It has been demonstrated that increased EAT thickness directly affects the coronary vessel wall, either through the secretion of inflammatory adipokines or via the vasa vasorum [1]. Clinical studies have reported that EAT is associated with the presence and severity of coronary artery disease and may represent an independent risk factor [17]. Moreover, because epicardial adipose tissue exhibits regional variability in its distribution, several studies have investigated which measurement site most accurately predicts coronary artery disease [18].

Epicardial adipose tissue can be assessed noninvasively using echocardiography, magnetic resonance imaging (MRI) or computed tomography (CT) [16]. While echocardiography has limitations due to observer dependency and the ability to obtain measurements from only restricted anatomical regions, MRI, although highly accurate, is limited by issues of accessibility, cost and contraindications related to implanted devices. In contrast, computed tomography is widely used in clinical practice because of its high spatial resolution, excellent tissue discrimination, and reliable measurement capability [1].

The availability of preoperative thoracic CT scans obtained during preoperative evaluation at our institution allows EAT assessment without the need for any additional procedures during surgical preparation. Therefore, the present study aimed to investigate the association between preoperative EAT thickness measured by CT and postoperative morbidity and mortality in patients undergoing isolated CABG and to evaluate the potential role of EAT thickness as an imaging-based risk marker for early postoperative complications. However, the current evidence base regarding the independent prognostic value of epicardial adipose tissue thickness in patients undergoing isolated CABG is limited and heterogeneous. Clarifying this potential association could improve risk stratification in the perioperative setting.

## 2. Materials and Methods

Medical records of patients who underwent isolated CABG at the Department of Cardiovascular Surgery, Kocaeli University Research and Application Hospital, between 1 January 2019 and 2 January 2023, were retrospectively reviewed. The study was a retrospective single-center observational cohort study. Ethical approval was obtained from the Kocaeli University Non-Interventional Clinical Research Ethics Committee (approval date: 9 February 2023; decision number: GOKAEK-2023/03.11).

At our institution, preoperative thoracic computed tomography is incorporated into the assessment of patients undergoing CABG surgery as part of surgical planning. In routine clinical practice, CT imaging is systematically performed in patients aged ≥65 years, whereas in younger patients it is obtained based on clinical indication. As a tertiary referral centre managing a high-risk population with multiple comorbidities, thoracic CT is commonly used to evaluate aortic calcification, pulmonary pathology and other thoracic conditions that may influence operative strategy and perioperative risk assessment. For the purposes of the present study, only patients with technically adequate thoracic CT imaging performed within three months prior to surgery were included to allow reliable measurement of epicardial adipose tissue.

Inclusion criteria:

(1) Patients who underwent isolated coronary artery bypass grafting;

(2) Availability of technically adequate thoracic computed tomography imaging in the institutional database performed within three months prior to surgery.

Exclusion criteria:

(1) Patients who underwent additional cardiac interventions for other cardiac pathologies identified during routine preoperative evaluations in addition to CABG;

(2) Patients who underwent concomitant vascular procedures in addition to CABG (including carotid endarterectomy, ascending aortic replacement, or femoropopliteal bypass grafting);

(3) History of previous cardiac surgery with pericardial opening;

(4) Left ventricular ejection fraction < 40% on preoperative echocardiography;

(5) History of myocardial infarction within the preceding 90 days;

(6) Emergency CABG performed within 24 h after coronary angiography due to symptomatic and hemodynamic instability;

(7) Presence of a preoperatively inserted intra-aortic balloon pump at the time;

(8) Preoperative serum creatinine level > 1.2 mg/dL and an estimated glomerular filtration rate < 60 mL/min/1.73 m^2^;

(9) Presence of atrial fibrillation on preoperative electrocardiographic evaluation or a history of atrial fibrillation;

(10) Presence of peripheral arterial disease requiring intervention and carotid artery stenosis >50%;

(11) History of cerebrovascular events.

We excluded patients with chronic kidney disease to minimise the confounding effect of pre-existing renal dysfunction on postoperative acute kidney injury outcomes and inflammatory status, which could potentially obscure the relationship between epicardial adipose tissue thickness and postoperative complications.

Between January 2019 and January 2023, discharge summaries, clinical outcomes, and imaging data of patients who underwent isolated coronary artery bypass grafting were retrospectively reviewed using the institutional hospital database (Nucleus) and the Picture Archiving and Communication System (PACS) at Kocaeli University Research and Application Hospital. Demographic characteristics, including age, sex, body mass index (BMI), smoking status, and comorbid conditions, were obtained from electronic medical records. Preoperative laboratory parameters included hemoglobin, leukocyte and neutrophil counts, platelet count, creatinine, urea, albumin, C-reactive protein, glycated hemoglobin (HbA1c), liver enzymes (AST and ALT), lipid profile (LDL, HDL, VLDL, total cholesterol, triglycerides), and troponin levels.

Preoperative chest radiographs, electrocardiographic rhythm assessment, and left ventricular ejection fraction values obtained by echocardiography were retrieved from institutional records. Operative reports and consultation notes were reviewed to determine additional comorbidities and surgical characteristics, including procedure type and duration. The European System for Cardiac Operative Risk Evaluation II (EuroSCORE II), a validated risk model widely used to estimate perioperative mortality risk in cardiac surgery, was calculated for all patients to assess baseline operative risk [19].

All surgical procedures were performed via median sternotomy under general anesthesia. The conduits selected for each patient included the internal mammary artery, the radial artery and/or the saphenous vein graft. Standard cardiopulmonary bypass (CPB) was established using aortic and right atrial cannulation following adequate systemic heparinization. During CPB, flow, arterial pressure, temperature and hematocrit were maintained within the institutional target ranges. Moderate hypothermia was induced and myocardial protection was achieved using intermittent antegrade hypothermic blood cardioplegia following aortic cross-clamping. Distal and proximal coronary anastomoses were performed using standard techniques. After completion of revascularization, patients were weaned from CPB, and the effects of heparin were reversed using protamine sulphate.

In patients undergoing off-pump CABG, procedures were performed via median sternotomy after conduit harvesting. Systemic heparinization was administered and proximal aortic anastomoses were performed using a side clamp. Cardiac positioning and stabilization were achieved using commercially available tissue stabilizer and positioning systems to allow exposure of the target coronary vessels. Distal coronary anastomoses were then completed under stabilized beating-heart conditions. After confirming graft patency and hemostasis, the procedure was completed according to standard surgical practice by reversing the heparin with protamine sulphate. Postoperatively, all patients are transferred to the intensive care unit. All surgical procedures in the patients included in the study were performed by the same team.

Preoperative non-contrast thoracic CT images of all patients were retrospectively reviewed using the institutional PACS to measure EAT thickness. All CT examinations were performed using a multidetector scanner with standardized acquisition parameters (detector collimation 64 × 0.5 mm, tube voltage 120 kVp, automatic tube current up to 400 mA, and gantry rotation time 500 ms). Axial images with 1 mm slice thickness and multiplanar reformatted images were used for evaluation.

**Figure 1:**
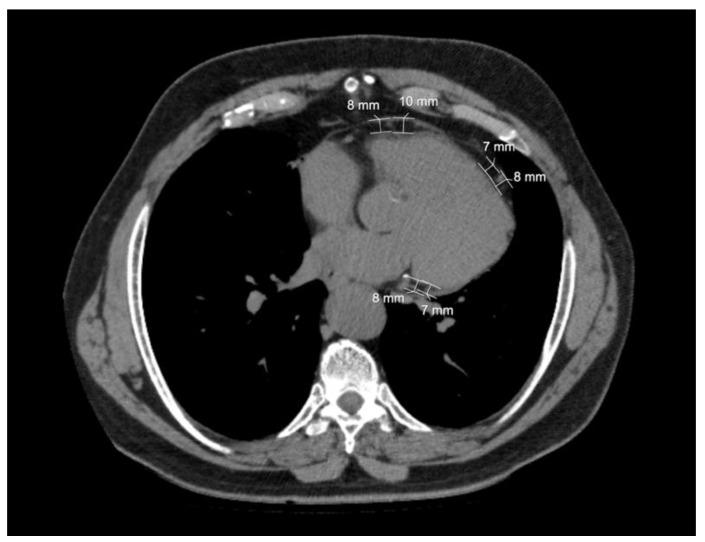
Measurement of epicardial adipose tissue thickness on axial thoracic computed tomography images. Special attention should be paid to ensuring that the measurement regions correspond specifically to perivascular adipose tissue.

Epicardial adipose tissue thickness was measured in three predefined anatomical regions of the heart: the anterior surface of the right ventricle along the course of the right coronary artery, the anterior interventricular septum, and the posterior wall (see Figure 1). Two measurements were obtained from each region, resulting in six individual measurements per patient. Total EAT thickness was then calculated as the mean of these values. These anatomical regions were specifically selected from perivascular (pericoronary) adipose tissue, in accordance with the methodology described by Gorter et al. [20]. For the analysis of right ventricular EAT thickness, the mean value of the four measurements obtained from the pericoronary regions over the anterior right ventricular surface and the anterior interventricular septum was used. Thus, two parameters were derived for analysis: total EAT thickness and right ventricular EAT thickness.

All epicardial adipose tissue measurements were performed retrospectively on preoperative CT scans by a single physician experienced in cardiovascular imaging using a predefined standardized measurement protocol. The observer performed all measurements based solely on CT images and without access to postoperative outcomes or clinical complication data at the time of image assessment.

Measurements were obtained on axial images with 1 mm slice thickness using the institutional PACS viewer under standard mediastinal window settings, without additional manual adjustment of window level or width, thereby ensuring consistency across all evaluations. To further support measurement reliability, epicardial adipose tissue thickness measurements were repeated in a subset of randomly selected scans by the same observer, and the results were confirmed to be consistent.

Atrial fibrillation is a supraventricular tachyarrhythmia characterized by uncoordinated atrial electrical activation and ineffective atrial contraction. According to the 2020 European Society of Cardiology (ESC) guidelines, diagnosis requires documentation on a 12-lead electrocardiogram or rhythm monitoring lasting at least 30 s [21].

Patients with atrial fibrillation identified on retrospective review of preoperative electrocardiograms (ECGs) or with a documented history of AF were excluded from the study. For patients included in the study, postoperative in-hospital and follow-up ECG and Holter recordings obtained within the first 6 months after discharge were evaluated.

Postoperative AF was defined as newly developed AF after surgery and classified according to ESC definitions as paroxysmal AF if it terminated spontaneously or with intervention within 7 days of onset, and as persistent AF if it lasted longer than 7 days as a sustained and continuous episode [21].

Postoperative acute kidney injury (AKI) was evaluated by reviewing serum creatinine values obtained during the first 7 postoperative days and classified according to established AKI diagnostic criteria. The diagnosis of AKI was based on the criteria defined by the Kidney Disease Improving Global Outcomes (KDIGO) guidelines which are presented in (Table 1) [22]. AKI was defined as an increase in serum creatinine ≥ 0.3 mg/dL within 48 h or ≥1.5 times baseline within 7 days, in accordance with KDIGO criteria.

Baseline serum creatinine levels of the patients included in the study were defined as the most recent concentration measured within one week prior to surgery, whereas postoperative serum creatinine levels were measured daily following the operation. Urine output was not used as a diagnostic criterion, as it may be influenced by diuretics and/or intravenous fluids administered during and after the CABG procedure [23,24].

Postoperative infection was evaluated based on the presence of microbial growth in wound, urine, sputum and tracheal aspirate cultures obtained during the in-hospital postoperative period.

Hospital electronic records were retrospectively reviewed, and discharge summaries were used to obtain data on length of hospital stay and intensive care unit stay, operative duration, CPB time, and aortic cross-clamp time. Postoperative outcomes including arrhythmia development, requirement for permanent pacing, inotropic support, intra-aortic balloon pump support, postoperative drainage volume, and the need for surgical revision were also recorded. In addition, data regarding the development of acute kidney injury and cerebrovascular events during the postoperative period were collected. All data were entered into IBM SPSS Statistics version 23 (IBM Corp., Armonk, NY, USA) for analysis.

Artificial intelligence-assisted language tools were only used for editing the English and improving clarity during the preparation of the manuscript. No AI tools were used for data analysis, interpretation or the generation of scientific results. All final content and conclusions were reviewed and approved by the authors.

## 3. Statistical Analysis

Statistical analyses were performed using IBM SPSS Statistics version 23 (IBM Corp., Armonk, NY, USA). Continuous variables were expressed as mean ± standard deviation or median (interquartile range) depending on distribution, and categorical variables as counts and percentages. Group comparisons were performed using Student’s *t*-test or Mann–Whitney U test for continuous variables and the chi-square test for categorical variables. Correlations between continuous variables were assessed using Spearman’s rank correlation analysis when appropriate.

Binary logistic regression analysis was used to evaluate the association between epicardial adipose tissue thickness parameters and postoperative complications. Outcomes (postoperative AF and AKI) were coded as binary variables (0 = absence, 1 = presence). Clinically relevant variables were entered simultaneously into the multivariable models, and odds ratios (ORs) with 95% confidence intervals (CIs) were reported. Results of the multivariable models were graphically presented using forest plots. Receiver operating characteristic (ROC) curve analysis was used to assess discrimination performance. Intraobserver reproducibility of epicardial adipose tissue measurements was evaluated using the intraclass correlation coefficient (ICC) and coefficient of variation (CV). A two-sided *p* value < 0.05 was considered statistically significant.

## 4. Results

Between 1 January 2019 and 2 January 2023, a total of 1061 patients underwent open-heart surgery at the Department of Cardiovascular Surgery, Kocaeli University Research and Application Hospital, of whom 674 underwent isolated CABG. Among these patients, those with available preoperative thoracic CT performed within three months prior to surgery were identified. After applying the predefined exclusion criteria, 100 eligible patients with technically adequate CT imaging were included in the final analysis. Baseline demographic and clinical characteristics of the study population are summarized in Table 2.

When the data were analyzed according to preoperative variables, the mean total EAT thickness was 5.65 ± 1.64 mm in women and 5.04 ± 1.12 mm in men. Total EAT thickness was significantly higher in women than in men (*p* = 0.04). A statistically significant association was observed between mean total EAT thickness and BMI categories; total EAT thickness was significantly lower in patients with normal BMI compared with those with class II obesity (*p* = 0.002).

Among the patients included in the study, 68 underwent surgery with CPB and 32 underwent off-pump procedures. No statistically significant association was observed between the surgical approach and the need for inotropic support, infection, development of paroxysmal AF, revision surgery, AKI, cerebrovascular events, or mortality (*p* > 0.05).

**Figure 2:**
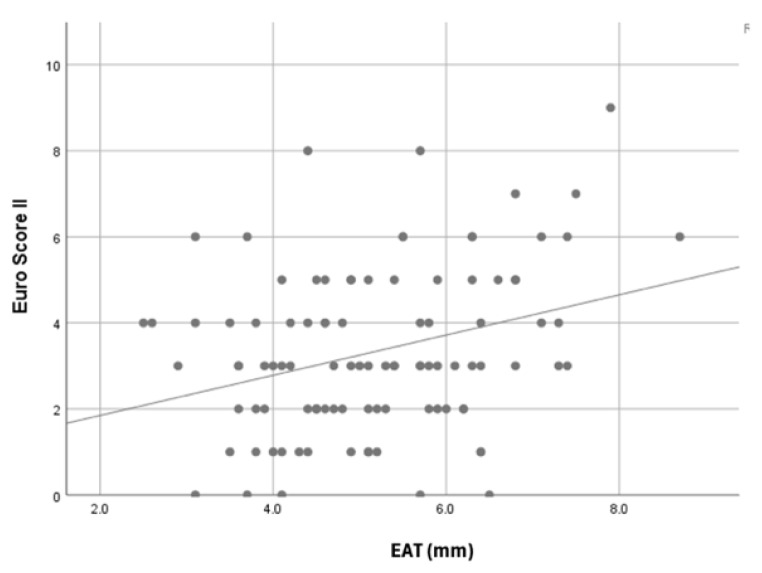
Scatter plot demonstrating the correlation between total epicardial adipose tissue thickness and EuroSCORE II. The fitted line represents the linear trend.

A statistically significant association was observed between total EAT thickness and EuroSCORE II (*p* = 0.008) (see Figure 2). When patients were categorized according to Euro- SCORE II risk groups, 35 were classified as low risk, 51 as intermediate risk, and 14 as high risk. Total EAT thickness was significantly associated with EuroSCORE II, and values were greater in high-risk patients compared with those in the low-risk and intermediate-risk groups (*p* = 0.004).

The incidence of postoperative complications is summarized as follows: paroxysmal AF occurred in 23 patients (23%), acute kidney injury in 18 (18%), postoperative infection in 3 (3%), revision surgery in 5 (5%), and inotropic support in 32 (32%). Persistent AF, cerebrovascular events, and in-hospital mortality were rare (1%, 1%, and 2%, respectively).

Total EAT thickness was significantly higher in patients who developed post operative paroxysmal atrial fibrillation compared with those who did not (*p* = 0.010). In addition, total EAT thickness was significantly greater in patients who developed postoperative in-hospital infections than in those without infection (*p* = 0.023) (Table 3). A statistically significant association was also observed between right ventricular EAT thickness and the development of postoperative persistent atrial fibrillation (*p* = 0.040).

Total EAT thickness did not differ significantly according to the need for inotropic support, postoperative persistent AF, cerebrovascular events, or AKI (*p* > 0.05). Similarly, no significant differences in total EAT thickness were observed with respect to postoperative bleeding or sternal revision (*p* > 0.05). Total EAT thickness was also not associated with intensive care unit stay or total hospital length of stay (*p* > 0.05). Finally, no significant difference in total EAT thickness was observed according to mortality status (*p* > 0.05) (Table 3).

Right ventricular EAT thickness did not differ significantly according to postoperative inotropic requirement, cerebrovascular events, or postoperative bleeding or sternal revision (*p* > 0.05). Likewise, no significant association was observed between right ventricular EAT thickness and postoperative mortality (*p* > 0.05).

Right ventricular EAT thickness was significantly associated with postoperative AKI (*p* < 0.001). When evaluated according to AKI stage, right ventricular EAT thickness was significantly lower in patients without postoperative AKI than in those who developed Stage 1 AKI (*p* = 0.001). In contrast, total EAT thickness did not differ significantly according to postoperative AKI status (*p* > 0.05) (Table 4).

Multivariable binary logistic regression analyses were performed to evaluate factors associated with postoperative atrial fibrillation and acute kidney injury. Clinically relevant perioperative variables (age, EuroSCORE II, diabetes mellitus, cross-clamp time, and epicardial adipose tissue thickness parameters) were entered simultaneously into the models (Table 5). For postoperative atrial fibrillation, age (OR 1.16, 95% CI 1.03–1.31, *p* = 0.018) and total EAT thickness (OR 1.74, 95% CI 1.10–2.76, *p* = 0.018) remained associated with the outcome after adjustment. For postoperative acute kidney injury, right ventricular EAT thickness was associated with the outcome in multivariable analysis (OR 2.03, 95% CI 1.31–3.15, *p* = 0.002).

**Figure 3:**
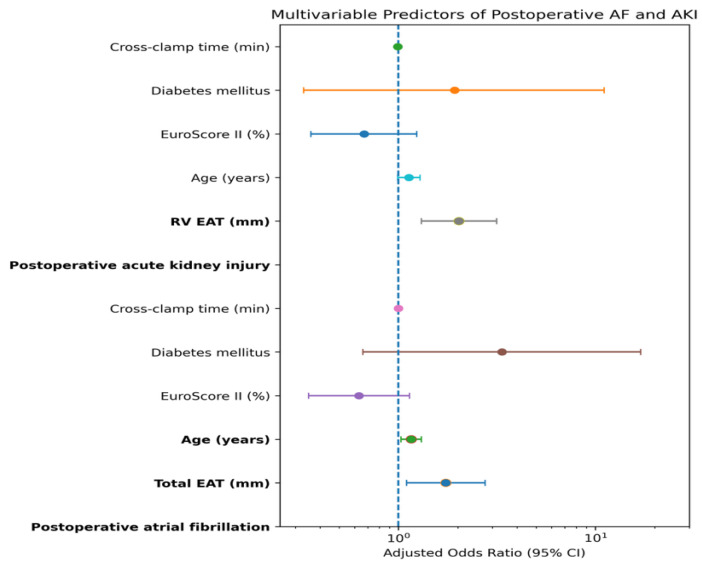
Forest plot showing adjusted odds ratios from multivariable logistic regression analyses for postoperative atrial fibrillation and acute kidney injury. Points indicate adjusted odds ratios and horizontal lines represent 95% confidence intervals (log scale).

Multivariable logistic regression analyses were performed to identify independent factors associated with postoperative complications. Increased total epicardial adipose tissue thickness was associated with a higher likelihood of postoperative AF, whereas increased right ventricular epicardial adipose tissue thickness was associated with postoperative AKI. Other clinical variables, including age, EuroSCORE II, diabetes mellitus, and cross-clamp time, were not independently associated with these outcomes in the adjusted models. The adjusted odds ratios with 95% confidence intervals are presented in Figure 3.

Receiver operating characteristic (ROC) curve analysis was performed to evaluate the discriminatory performance of epicardial adipose tissue thickness parameters for postoperative complications. Total EAT thickness demonstrated modest discrimination for postoperative paroxysmal atrial fibrillation (AUC 0.69), with an optimal threshold value of 4.95 mm (sensitivity 82.6%, specificity 55.8%). Right ventricular EAT thickness showed acceptable discrimination for postoperative acute kidney injury (AUC 0.80), with an optimal threshold value of 7.15 mm (sensitivity 77%, specificity 82%).

An intraobserver reproducibility analysis was performed in 20 randomly selected patients using blinded repeated measurements. Agreement was moderate for both total and right ventricular epicardial adipose tissue thickness measurements, with ICC values of 0.65 and 0.70 and coefficients of variation of 11.2% and 10.3%, respectively.

No association was identified between preoperative total or right ventricular EAT thickness and the development of inotropic support requirement, cerebrovascular events, bleeding, or sternal revision following isolated CABG. Likewise, no association was observed between postoperative mortality and either total or right ventricular EAT thickness; this finding may reflect the limited sample size and the low number of mortality events.

## 5. Discussion

In this study, the impact of preoperative epicardial adipose tissue thickness measured by computed tomography on postoperative morbidity and mortality in patients undergoing isolated coronary artery bypass grafting was investigated. Total EAT thickness was found to be significantly associated with paroxysmal atrial fibrillation and in-hospital infection, whereas right ventricular EAT thickness was significantly associated with acute kidney injury and postoperative persistent atrial fibrillation.

In recent years, the need for novel biochemical, imaging-based, and analytical biomarkers for the early detection and risk stratification of cardiovascular diseases has increased substantially. Multiple studies have demonstrated that epicardial adipose tissue thickness may serve as an independent risk factor for coronary artery disease and has the potential to reflect both the severity and extent of the disease [17]. However, although previous studies have explored the association between EAT and cardiovascular outcomes, including postoperative atrial fibrillation, data specifically evaluating preoperative computed tomography-based epicardial adipose tissue thickness in relation to multiple early postoperative complications following isolated CABG remain limited.

The pathophysiological role of epicardial adipose tissue in coronary artery disease is increasingly recognised, leading to increased interest in imaging-based assessment of EAT thickness. Early studies used echocardiography; for instance, Jeong et al. [25] demonstrated an association between EAT thickness and well-established coronary artery disease risk factors. Nevertheless, echocardiographic evaluation may be constrained by restricted acoustic windows and interobserver variability, as reported by Saura et al. [26], emphasising the limitations of this modality for comprehensive EAT assessment. CT allows more comprehensive visualisation of the epicardial fat depot. Although CT-based volumetric assessment provides detailed information, it requires additional software and time-consuming image processing. Wang et al. [18] reported that regional EAT thickness measurements showed stronger associations with coronary artery disease than volumetric assessment. Therefore, in the present study, EAT thickness was measured using thoracic CT due to its reliability, practicality and wider clinical availability. Volumetric assessment was not performed due to the limitations of non-contrast thoracic CT. Furthermore, as CT imaging had already been performed for routine clinical evaluation, no additional radiation exposure was incurred for the purposes of this study.

When patients were evaluated using EuroSCORE II, total epicardial adipose tissue thickness was significantly higher in high-risk patients compared with those in the low- and intermediate-risk groups, whereas no association was observed for right ventricular EAT thickness. Previous studies have shown that increased epicardial adipose tissue is associated with greater coronary disease burden and adverse cardiovascular outcomes, suggesting that EAT may reflect overall inflammatory and cardiovascular risk status. Although established clinical risk scores such as EuroSCORE II are widely used for perioperative risk stratification, imaging-based parameters such as epicardial adipose tissue thickness may provide complementary information by reflecting local inflammatory and metabolic status not fully captured by traditional clinical variables. Therefore, EAT thickness should be considered a potential adjunctive risk marker rather than a replacement for existing scoring systems.

Evidence from studies investigating epicardial adipose tissue indicates that its local and systemic biochemical effects are closely linked to the heart’s anatomical proximity and clinical function. Three principal mechanisms have been proposed to explain the association between epicardial adipose tissue and atrial fibrillation: its interaction with autonomic ganglia, its production of inflammatory adipokines, and its anatomical proximity to the atria [27]. Autonomic ganglionated plexuses are located in close proximity to epicardial adipose tissue and can be influenced by locally secreted mediators derived from adipose tissue. Studies have demonstrated that stimulation of these autonomic ganglionated plexuses results in marked shortening of action potential duration as well as transient increases in intracellular calcium within the pulmonary veins and atrial myocardium, thereby potentially contributing to the initiation and perpetuation of atrial fibrillation [28]. Inflammatory cytokines such as interleukin-6 (IL-6) and tumor necrosis factor-α (TNF-α) have been shown to play an important role in the pathogenesis and clinical course of atrial fibrillation [29]. The incidence of postoperative atrial fibrillation has been found to peak on postoperative days 2 and 3, coinciding with the time at which serum C-reactive protein levels reach their maximum [30]. Because of its close anatomical proximity to the atrial wall, epicardial adipose tissue may exert direct atrial arrhythmogenic effects [31]. In a study by Batal et al. [32], increased posterior left atrial fat thickness was found to be associated with atrial fibrillation burden independent of age, BMI, and left atrial area. Shirani et al. [33] reported that excessive fat accumulation within the atrial septum was associated with a higher incidence of atrial arrhythmias. In our study, total EAT thickness measured by preoperative computed tomography was found to be associated with paroxysmal atrial fibrillation following CABG; total EAT thickness was significantly greater in patients who developed postoperative paroxysmal AF compared with those who did not (*p* = 0.010). During follow-up, atrial fibrillation was reversible in 96% of patients who developed postoperative paroxysmal AF.

In our study, multivariable logistic regression analyses indicated that total epicardial adipose tissue thickness was associated with postoperative paroxysmal atrial fibrillation, while right ventricular epicardial adipose tissue thickness showed an association with postoperative acute kidney injury following isolated CABG (Table 5).

Sternal wound infections may also develop secondary to postoperative complications and can prolong hospital length of stay. Although the incidence of sternal wound infections has declined to approximately 1–4% of all cardiac surgical procedures, they remain associated with increased morbidity, mortality, and reduced long-term survival [34]. In our study, postoperative in-hospital infections occurred in 3% of patients and were identified as being of respiratory origin; no sternal wound infections were observed. When the relationship between preoperative total EAT thickness and the development of postoperative in-hospital infection was evaluated, total EAT thickness was significantly greater in patients who developed infection (*p* = 0.023).

In our cohort, postoperative acute kidney injury developed in 17% of patients. Among those who developed AKI, 59% had undergone surgery with CPB and 41% an off-pump procedure; however, surgical technique was not associated with postoperative AKI (*p* > 0.05). This finding may reflect the persistence of inflammatory injury related to surgical trauma even in off-pump procedures. Importantly, right ventricular EAT thickness measured preoperatively by computed tomography showed a strong association with postoperative AKI (*p* < 0.001). RV EAT thickness was significantly lower in patients without AKI compared with those who developed postoperative Stage 1 AKI, whereas total EAT thickness showed no association with AKI following isolated CABG. Previous studies have suggested that epicardial adipose tissue reflects systemic inflammatory burden and cardiovascular risk status, which may contribute to postoperative organ dysfunction, including renal injury [14,35]. This pathophysiological relationship may explain the observed association between RV EAT thickness and postoperative AKI in the present study.

Our study has several limitations. The principal limitations include its single-center design, the relatively small sample size, and the retrospective nature of the study. Because of the limited sample size and number of outcome events, formal evaluation of incremental predictive performance beyond established surgical risk scores was not performed and should be addressed in future larger prospective studies. Patients undergoing both on-pump and off-pump bypass procedures were included in our study, resulting in a heterogeneous cohort; however, our analyses demonstrated no association between the use of on-pump versus off-pump techniques in isolated CABG and the development of postoperative paroxysmal AF. The generalizability of the present findings to patients with impaired renal function may be limited, because patients with baseline chronic kidney disease were excluded in order to reduce the potential confounding effect of pre-existing renal dysfunction on postoperative outcomes. Another limitation was that routine thoracic-CT examinations at our institution are performed without contrast enhancement; therefore, all measurements in the included patients were derived from non-contrast CT images. Owing to the absence of contrast, focal measurements of EAT thickness around culprit lesions could not be performed. Because epicardial adipose tissue measurements were performed retrospectively by a single experienced observer using a standardized protocol and consistent CT viewing settings, measurement variability was minimized; however, formal interobserver reproducibility analysis was not performed and should be evaluated in future prospective studies. The relatively small number of events for some complications, particularly persistent atrial fibrillation and infection, should be considered when interpreting the statistical findings. Finally, some complication subgroups contained very small numbers of events, which may limit the statistical power of subgroup comparisons. The small number of mortality events (*n* = 2) may have limited the statistical power to evaluate a potential association between EAT thickness and mortality. These findings should be interpreted within the inherent limitations of a retrospective observational study conducted in a selected patient population. Therefore, the present findings should be interpreted with caution and considered exploratory and hypothesis-generating rather than definitive. Larger, preferably multicenter prospective studies are warranted to confirm the magnitude and direction of these associations before firm clinical implications can be established.

## 6. Conclusions and Recommendations

In this retrospective single-center study, increased preoperative epicardial adipose tissue thickness measured on thoracic computed tomography was associated with several early postoperative complications following isolated CABG, including atrial fibrillation, in-hospital infection, and acute kidney injury, suggesting a potential link between epicardial adipose tissue burden and perioperative risk profile. The observed association between total EAT thickness and EuroSCORE II further supports the potential role of EAT as a marker reflecting overall perioperative cardiovascular risk.

Preoperative EAT thickness measured by computed tomography may represent a potentially useful imaging-based risk marker for early postoperative complications following isolated CABG. However, given the retrospective design and limited sample size, these findings require validation in larger prospective multicenter studies.

## Figures and Tables

**Table 1:** Diagnostic criteria for acute kidney injury according to the Kidney Disease: Improving Global Outcomes (KDIGO) guidelines [22].

Stage	Serum Creatinine	Urine Output (U/O)
1	Increase in creatinine to 1.5–1.9 times baseline or an absolute increase of ≥26.5 µmol/L (0.3 mg/dL)	U/O < 0.5 mL/kg/h for 6–12 h
2	Increase in creatinine to 2.0–2.9 times baseline	U/O < 0.5 mL/kg/h for 12–24 h
3	Increase in creatinine to >3 times baseline; or creatinine ≥354 µmol/L (4.0 mg/dL) with an acute rise of ≥44 µmol/L (0.5 mg/dL); or initiation of renal replacement therapy	U/O < 0.3 mL/kg/h for >24 h; or anuria for ≥12 h

**Table 2:** Baseline demographic and clinical characteristics of the study population (*n* = 100).

		Mean ± SD	
Age (years)		65.55 ± 7.84	
BMI (kg/m^2^)		28.77 ± 4.54	
EuroScore II (%)		3.33 ± 1.91	
Cross-clamp time (min)		64.32 ± 20.04	
CPB time (min)		113.53 ± 31.42	
Total EAT thickness (mm)		5.17 ± 1.27	
Right ventricular EAT thickness (mm)		6.60 ± 3.80	
		*n*	(%)
Sex	Female	22	22
	Male	78	78
BMI (kg/m^2^)	Normal	20	20
	Overweight	48	48
	Obesity class I	21	21
	Obesity class II	11	11
Diabetes mellitus	No	47	47
	Yes	53	53
Hypertension	No	31	31
	Yes	69	69
Hyperlipidemia	No	33	33
	Yes	67	67
Chronic obstructive pulmonary disease	No	87	87
	Yes	13	13
Smoking status	No	47	47
	Yes	53	53
Peripheral arterial disease	No	93	93
	Yes	7	7
Carotid artery stenosis	No	91	91
	Yes	9	9
EuroScore II	Low risk (0–2)	35	35
	Intermediate risk (3–5)	51	51
	High risk (>6)	14	14

**Table 3:** Association between total EAT thickness and postoperative complications after CABG. Values are presented as mean ± SD. The number of patients in each subgroup is shown. Significant associations were observed for paroxysmal atrial fibrillation and postoperative infection.

Postoperative Complications			
	Total EAT (mm), Mean ± SD		*p* Value
Inotropic support requirement			
No (*n* = 68)	5.07	±1.25	0.157
Yes (*n* = 32)	5.38	±1.32	
Paroxysmal AF			
No (*n* = 77)	4.99	±1.25	**0** **.** **010**
Yes (*n* = 23)	5.76	±1.16	
Persistent AF			
No (*n* = 99)	5.14	±1.25	0.092
Yes (*n* = 1)	7.30	-	
Infection			
No (*n* = 97)	5.12	±1.23	**0** **.** **023**
Yes (*n* = 3)	6.80	±1.49	
Cerebrovascular event			
No (*n* = 99)	5.18	±1.27	0.358
Yes (*n* = 1)	4.00	-	
Acute kidney injury			
No (*n* = 82)	5.11	±1.23	0.300
Stage 1 (*n* = 17)	5.36	±1.44	
Stage 2 (*n* = 0)	-	-	
Stage 3 (*n* = 1)	7.10	-	
Re-exploration			
No (*n* = 95)	5.13	±1.28	0.371
Bleeding (*n* = 2)	5.95	±1.20	
Sternal (*n* = 3)	4.70	±0.34	
Mortality			
Survived (*n* = 98)	5.19	±1.28	0.305
Deceased (*n* = 2)	4.30	±0.28	

Bold values indicate statistically significant associations (*p* < 0.05).

**Table 4:** Association of total and right ventricular EAT thickness with postoperative acute kidney injury (AKI).

	Total EAT (mm)			Right Ventricular EAT (mm)		
AKI	Mean	SD	*p*	Mean	SD	*p*
No	5.11	1.23	0.587	5.82	1.54	**<0.001**
Yes	5.36	1.44		7.37	1.25	
	Total EAT (mm)			Right Ventricular EAT (mm)		
AKI	Mean	SD	*p*	Mean	SD	*p*
No	5.11	1.23	0.300	5.82	1.55	**0.001**
Stage 1	5.36	1.44		7.37	1.25	
Stage 2	-	-		-	-	
Stage 3	7.10	-		6.00	-	

Bold values indicate statistically significant associations (*p* < 0.05).

**Table 5:** Multivariable logistic regression analysis for postoperative complications.

Outcome	Variable	OR (Per Unit)	95% CI	*p* Value
Paroxysmal AF	Total EAT (mm)	1.74	1.10–2.76	**0.018**
	Age (years)	1.16	1.03–1.31	**0.018**
	EuroSCORE II	0.63	0.35–1.14	0.127
	Diabetes mellitus	3.35	0.66–17.00	0.145
	Cross-clamp time	1.00	0.99–1.02	0.795
Acute Kidney Injury	RV EAT (mm)	2.03	1.31–3.15	**0.002**
	Age (years)	1.13	0.99–1.29	0.063
	EuroSCORE II	0.67	0.36–1.24	0.198
	Diabetes mellitus	1.93	0.33–11.09	0.463
	Cross-clamp time	0.99	0.97–1.01	0.244

Bold values indicate statistically significant associations (*p* < 0.05). Binary logistic regression analysis was performed. Variables clinically relevant to perioperative risk were entered simultaneously into the multivariable model. AF: atrial fibrillation; AKI: acute kidney injury; EAT: epicardial adipose tissue.

## Data Availability

The data supporting the findings of this study are available from the corresponding author upon reasonable request. The data are not publicly available due to privacy and ethical restrictions.
